# Evaluating the effects of a temporary fostering program on shelter dog welfare

**DOI:** 10.7717/peerj.6620

**Published:** 2019-03-27

**Authors:** Lisa M. Gunter, Erica N. Feuerbacher, Rachel J. Gilchrist, Clive D.L. Wynne

**Affiliations:** 1 Department of Psychology, Arizona State University, Tempe, AZ, United States of America; 2 Department of Animal and Poultry Sciences, Virginia Polytechnic Institute and State University (Virginia Tech), Blacksburg, VA, United States of America

**Keywords:** Dogs, Animal shelter, Cortisol, Welfare, Human-animal interaction, Stress, Enrichment

## Abstract

One of the greatest stressors for dogs living in animal shelters is social isolation. Many studies have demonstrated that human interaction reduces cortisol in shelter dogs, with the possibility that longer periods of interaction may yield greater effects. These types of interventions are contingent upon removing the dog from the kennel and any such reductions in cortisol are often lost when the dog returns to the kennel. More recently, animal shelters are utilizing short-term fostering programs to provide relief from the perceived stresses of kennel life; however the effects of these programs are not well understood. This study assessed the impacts of one- and two-night fostering programs on the urinary cortisol levels, resting pulse rates, longest bout of uninterrupted rest, and proportion of time spent resting of dogs awaiting adoption. Five animal shelters, open and limited-admission facilities, from across the United States participated in the study. During the study, dogs’ urine was collected in the morning before, during, and after fostering stays for cortisol: creatinine analysis. Non-invasive health monitors were worn by the dogs, which collected heart rates and activity levels, in the shelter and in foster homes. In total, 207 dogs participated in the study, and 1,076 cortisol values were used in our analysis. Across all shelters, we found that dogs’ cortisol: creatinine ratios dropped significantly during their fostering stay, but returned to baseline levels after return to the shelter. However, the observed reduction in cortisol varied in magnitude across shelters. We found that dogs of greater weight, age, and average resting pulse rate had higher cortisol levels; and dogs with longer bouts of uninterrupted rest had lower cortisol levels. Dogs had their longest bouts of rest during sleepovers, followed by in the shelter after their sleepovers. Lastly, significant differences were found when comparing in-shelter cortisol values at our five shelters, differences that were in some cases greater than the impact of the fostering intervention itself. Considering the diversity of facilities that participated in this study, it is possible that as yet unstudied, shelter-specific, environmental factors could be contributing to the overall welfare of shelter dogs. Thus while a reprieve from the shelter is impactful for dogs awaiting adoption, mitigating the stressors present in kenneling conditions should also be addressed to improve the lives of shelter dogs.

## Introduction

Between 4–5.5 million dogs enter animal shelters annually in United States. Many will find new homes or return to their owners, but about 14% of these dogs will be euthanized (Woodruff & Smith, 2017; Rowan & Kartal, 2018). While the number of dogs arriving at animal shelters is declining as well as those that are ultimately euthanized, lengths of stay in the shelter are likely increasing as dogs await adoption (Protopopova, 2016). As such, shelters are becoming less like temporary ports in the storm for homeless pets and more akin to child orphanages (Barrett & Greene, 2015).

Within the novel environment of the shelter, dogs experience a variety of potential stressors upon entry and during their stay, including disruptive sounds, restriction of movement, and loss of social attachments (Taylor & Mills, 2007). Previous research has found that noise levels in shelter kennels can reach and exceed 100 dB (Sales et al., 1997; Coppola, Enns & Grandin, 2006; Scheifele et al., 2012; Venn, 2013), surpassing the 90 dB limit set for human exposure set by the Occupational Safety and Health Administration for an eight-hour period (United States Department of Labor, Occupational Health and Safety Administration, 1983). Scheifele et al. (2012) found that six months of exposure to noise at 100 dB and above at an animal shelter resulted in hearing loss for all dogs measured within the study.

Dogs in homes spend much of their day in sedentary activities and are active in moderate to vigorous physical exercise for small portions of the day (Morrison et al., 2013). However, the space allowed dogs in animal shelters is likely inadequate to meet their basic activity needs (Hubrecht, Wickens & Kirkwood, 1995). In addition to the inhibition of free movement due to kennel size, shelter living also limits dogs’ ability to interact with other dogs as they are often housed singly to prevent injury and disease (Hubrecht, 1995); yet this social isolation is likely detrimental to their welfare (Beerda et al., 1999a; Beerda et al., 1999b). It has been suggested that a stay at an animal shelter may be one of the most socially-isolating new experiences a companion dog may face in its lifetime (Hennessy et al., 1997).

Beyond the limitations of companionship, space and excessive noise, life in the shelter often results in dogs having little control over their daily lives, particularly contingencies surrounding interactions with other dogs and people (Hennessy et al., 1997). This lack of control may be a source of apathetic behavior, wherein dogs display less interest towards shelter visitors over time (Wells & Hepper, 1992) and remain in the back of the kennel (Wells, Graham & Hepper, 2002). Similarly, the lack of predictability within the shelter environment can also have a psychological impact, such as the uncertainty related to routines, like leashed walks or opportunities to eliminate outside of the kennel (Hennessy et al., 1998).

Cortisol is one of most widely used physiological markers of the stress response in dogs (Hewson, Hiby & Bradshaw, 2007). Previous studies comparing cortisol in owned and shelter dogs have found elevated levels for those living in shelter conditions (Sandri et al., 2015; Dudley, Schiml & Hennessy, 2015), while dogs kenneled for the first time experience three-fold increases in cortisol when entering kennels compared to at-home levels (Rooney, Gaines & Bradshaw, 2007). Welfare interventions carried out at the shelter have demonstrated that spending time with humans can reduce this measure of canine stress. Menor-Campos, Molleda-Carbonell & López-Rodríguez (2011) reported that just 30 min of walking and interaction decreased cortisol levels of shelter dogs and improved behavioral scores. Regardless of activity, be it petting, play, or simple presence in the same room, human interaction improved the welfare of shelter dogs when compared to remaining in or being removed from the kennel but without a person present (Shiverdecker, Schiml & Hennessy, 2013). While the benefits of these interventions, such as reductions in cortisol, are often lost when dogs return to their kennels, researchers have speculated that longer periods of interaction with people could be additionally beneficial for shelter dogs (Coppola, Grandin & Enns, 2006).

One intervention that would increase dogs’ time spent in interaction with humans is short-term fostering—defined as a temporary stay in a human home. However, little is known about how temporarily leaving the shelter environment could impact the welfare of dogs awaiting adoption. Prior studies investigating the effects of temporary and trial adoptions (Normando et al., 2006) as well as programs that utilize foster homes to facilitate adoption (Mohan-Gibbons et al., 2014) have found that dogs that participate in these programs are returned less frequently by their new owners. Additionally, adopters in Mohan-Gibbons et al. (2014) reported that they more often used information provided by foster families, as compared to shelter staff, in their adoption decision-making. While it is possible that time in a foster home could allow for more interaction with humans, the novelty of the environment and its potential stressors could reduce those benefits.

Beyond the physiological effects of stress that can be quantified through cortisol, measuring activity can be another component to shelter welfare assessment. Jones et al. (2014) found dogs’ maximum and average activity levels were related to increased salivary and urinary cortisol levels, respectively. Owczarczak-Garstecka & Burman (2016) observed that dogs in a rescue center slept for 45% of a 24-hour cycle whereas dogs in homes have been shown to rest for 60% of the day (Morrison et al., 2013). In the present study, we hypothesized that brief stays of one to two nights in a foster home would result in lower urinary cortisol: creatinine (C/C) ratios for dogs awaiting adoption as compared to ratios collected before or after their stays. Additionally, these dogs were predicted to show increased rest in the foster home compared to their time in the animal shelter.

## Materials & Methods

### Shelters

We collected data on the impacts of brief sleepovers at five US animal shelters: Best Friends Animal Sanctuary (BFAS; May–June 2016), Arizona Humane Society (AHS; February–March 2017), the Humane Society of Western Montana (HSWM; May 2017), DeKalb County Animal Services (DCAS; June 2017), and the Society for the Prevention of Cruelty to Animals of Texas (SPCATX; July 2017). The shelters varied in their geographical location, size, admission type, and annual dog intake (Table 1).

**Table 1:** Admission type and location of shelter, canine intake for prior year, and number of subject dogs and samples.

Shelter	Admission type and state	Prior year canine intake	Subject dogs	Complete sequences^a^	Samples collected	Samples removed^b^
BFAS	Limited, UT	743	39	38	131	3
AHS	Open, AZ	6,607	43	32	243	9
HSWM	Limited, MT	847	40	33	235	4
DCAS	Open, GA	5,686	43	41	254	8
SPCATX	Limited, TX	4,818	42	40	254	17

**Notes.**

a Complete sequences are dogs in which all experiment time-points were collected.

b Samples were removed from data analysis when C/C ratio values were three standard deviations above the shelter’s mean.

The staff at each shelter determined which dogs participated in the study, and foster homes met all shelter requirements prior to fostering. Thus, we did not intercede on organizational decisions regarding which dogs were temporarily fostered, requirements for volunteers fostering dogs, or the shelter’s expectations while dogs were in the care of fosterers. Shelter staff were observed to select dogs without histories of aggressive behavior so as to minimize the likelihood of injury or harm to the researchers and fosterers. Dogs that were fearful of the urine collection equipment were not included due to the inability of the researchers to collect samples needed for cortisol analysis.

### Sleepovers

Dogs were temporarily fostered for one (BFAS) or two nights (all other shelters). Foster volunteers picked up dogs from the shelter where staff provided them with fostering instructions and supplies. The authors discussed the purpose of the study with the foster volunteers as well as instructions for urine collection and completing a behavioral questionnaire. When the dogs returned to the shelter following the sleepover, the authors met with foster volunteers, collected urine samples and questionnaires, and dogs were returned to their kennels.

### Urine Collection

Urine was collected before, during, and after sleepovers for C/C analysis. For dogs at BFAS, the authors collected baseline and post-sleepover urine samples in the shelter the morning before and after, respectively, the dog was temporarily fostered. The fosterer collected the urine sample on the morning after the sleepover before returning the dog to the shelter. This resulted in three collection time-points for each dog at BFAS. For all other shelters, dogs had two days of the baseline, sleepover, and return-baseline phases. The authors collected urine the morning before the day of the sleepover and the morning prior to sleepover, as well as for two mornings after the dog returned from foster care. Fosterers collected urine the mornings after the first night and second night of the sleepover before returning the dog to the shelter. In total, six time-points were collected with each dog at AHS, HSWM, DCAS, and SPCATX.

Samples were collected using Olympic Clean-Catch™ plastic trays taped to 36-inch (91-cm) “Pickup and Reach” tools (Harbor Freight, Calabasas, CA, USA). After collection in the tray, samples were poured into 10 mL plastic conical bottom centrifuge tubes with screw caps for storage. Collection trays were rinsed with water prior to use. Fosterers were provided with urine collection kits, including a “Pickup and Reach” tool with taped plastic trays and labeled storage tubes.

For shelter urine collection pre- and post-sleepover, the authors removed dogs from the kennel and walked them on-leash to an approved outdoor location for elimination. After urine collection, dogs were returned to their kennels. Urine samples were collected between 7:00 am and 9:30 am, immediately placed in a cooler with ice, and in a freezer within two hours at a temperature of −18 °C. Five percent of samples fell outside this collection range due to dogs not urinating when walked or not providing an adequate volume of urine (minimum 1.5 mL). In these cases, dogs remained with the authors and were provided a mixture of wet food and water before another attempt at urine collection was made. Foster volunteers were instructed to collect urine upon the dog’s waking when given their first elimination opportunity. Urine samples brought to the shelter by fosterers within 30 min of collection were typically not cooled or frozen until arrival at the shelter. Fosterers whose samples arrived 30 mins after collection (i.e., first morning collection at AHS, HSWM, DCAS, and SPCATX) were instructed to freeze these samples until transport to the shelter. As with shelter urine collection, storage tubes were immediately placed in a cooler with ice, then placed in a freezer until shipment at a temperature of −18 °C. Time of collection was noted for each sample.

Frozen urine samples were shipped overnight on dry ice to Animal Reference Pathology (Salt Lake City, UT, USA) for C/C analysis. Analysis occurred within one month of sample collection. Analysis was conducted using an automated wet biochemistry analyzer (Dimension Xpand Plus; Siemens Healthcare Diagnostics Inc., Newark, DE, USA) for the measurement of creatinine. Human (Urine) Precision Control 2 and 3 Chemistry Controls (Control level 2 #UN1557, Control level 3 #UE1558; Randox Laboratories Limited, Crumlin, County Antrim, UK) were run on each day of testing urine samples. These two levels of control were reconstituted and stored according to manufacturer instructions.

Cortisol was measured using a commercially available product designed for an enzyme-amplified chemiluminescence assay system (Immulite 1000; Diagnostic Products Corporation, Los Angeles, CA, USA). Cortisol: creatinine ratios (measured in µmol/l: µmol/l) × 10^−6^ were then calculated.

### Health-monitoring collars

At AHS, HSWM, DCAS, and SPCATX, health-monitoring collars (PetPace, Shefayim, Israel) collected dogs’ temperature, pulse, respiration, activity, and positions through the use of acoustics, 3-D accelerometer, and thermistors. Once collected, data was transmitted via ultra high frequency radio to nearby internet-connected base units, which sent information to PetPace online data portals for analysis.

Physical activity captured by PetPace collars has been found to strongly correlate with data from Actigraph accelerometers, a previously validated device used to measure canine activity (Belda et al., 2018). Additionally, measures of pulse and respiration collected by PetPace collars have been shown to correspond positively to increasing levels of activity in dogs (Ortmeyer, Robey & McDonald, 2018). For the purposes of this study, average pulse rates while the dogs were at rest (as determined by the collar’s activity measurement), longest bout of uninterrupted rest, and proportion of resting activity (out of total activity measured) for the three phases (before, during, and after sleepover) were calculated for each dog.

### Statistical analysis

Using data collected at BFAS, multiple linear regression analyses with backward elimination were employed to determine whether urinary C/C values could be predicted from variables related to the dog as well as variables associated with the BFAS sleepover program. To test whether these cortisol values changed between collection times, we analyzed C/C ratios with a linear mixed model. Dog and intercept were entered as random effects with time-point and covariates included as fixed effects in the model. Restricted maximum likelihood estimation (REML) and diagonal covariance matrices were used.

With cortisol and creatinine analyses conducted on the urine samples of dogs from the remaining four shelters (AHS, HSWM, DCAS, and SPCATX), values for the six time-points were utilized in the statistical analyses. Individually with each shelter’s data, multiple linear regression analyses with backward elimination were conducted to determine whether urinary C/C values could be predicted from our dog variables, including data from the health-monitoring collars. To examine whether cortisol values changed from the shelter and foster home, we analyzed C/C ratios with a linear mixed model. Dog and intercept were entered as random effects with time-point and covariates included as fixed effects in the model. REML and diagonal covariance matrices were used. Linear mixed modeling was conducted with the data from each site and in with all shelters. Quartile-quartile plots were used to assess the normality of the data obtained from all shelters against the standard normal distribution prior to analysis.

In our multi-shelter analyses, data from AHS, HSWM, DCAS, and SPCATX were used in a multiple linear regression analysis with backward elimination to uncover whether C/C values could be predicted from dog and health-monitoring variables. Phase variables, created when data was collected at two consecutive time-points that were either before, during or after the sleepover, include mean C/C, LOS, and resting pulse values.

Data from all five study sites were utilized in a linear mixed model analysis to determine whether shelter differences could be detected from in-shelter cortisol values obtained from collections taken before and after sleepover. To determine whether dogs’ longest bout of rest and mean resting pulse differed before, during, and after sleepover and whether a shelter or shelter-by-time-point interaction could be detected, phase data collected from the dogs’ health-monitoring collars at AHS, HSWM, DCAS, and SPCATX were used in a linear mixed model analysis. In both models, dog and intercept were entered as random effects with shelter and covariates included as fixed effects in the model. REML and diagonal covariance matrices were used. In all linear mixed models where post-hoc comparisons were conducted, a Sidak correction was utilized to allow for multiple comparisons.

Thus, statistical analyses were conducted with each shelter’s data separately, averaging across the before, during, and after sleepover phases; and with separate and phase data across multiple shelters to understand the relationship of various dog and shelter variables to urinary C/C values.

### Ethical statement

The Carroll College Institutional Animal Care and Use Committee approved procedures carried out at BFAS (IACUC: CC0009). Procedures carried out at AHS, HSWM, DCAS, and SPCATX were approved by the Arizona State University Institutional Care and Use Committee (IACUC: 17-1552R).

## Results

### Best Friends Animal Sanctuary

Descriptive statistics describing BFAS and the dogs that participated in the study are included in Tables 1 and 2. Additionally because of the shelter’s pre-existing sleepover program, dogs at BFAS had previously participated in its program an average 18 times prior to the study (*SD* 21.32). A median of 13.18 days (*SD* 12.02) had passed since the dog’s last sleepover. Most dogs in the study were taken off-site during their sleepover (73.20%).

**Table 2:** Shelter mean of participating dogs’ sex, LOS, age, weight, and cortisol: creatinine ratio values.

Shelter	Sex %	LOS (days)		Age (mths)		Weight (kg)		Cortisol: creatinine ratio (×10^−6^)	
		*M*	*SD*	*M*	*SD*	*M*	*SD*	*M*	*SD*
BFAS	M: 61.9	463.5	594.5	63.6	42.5	24.4	5.7	22.0	7.2
AHS	M: 67.4	14.1	9.3	49.0	32.9	19.2	6.0	39.1	17.7
HSWM	M: 53.5	9.7	12.1	32.7	33.4	16.7	11.3	29.2	11.0
DCAS	F: 60.5	59.4	57.4	31.9	23.1	21.4	5.4	26.6	11.8
SPCATX	F: 51.2	22.2	1.5	55.5	28.8	21.2	9.0	22.8	9.9

Multiple linear regression analyses with backward elimination were used to identify whether sex, age, weight, length of stay (LOS), number of previous sleepovers, days since last sleepover, or location of sleepover significantly predicted C/C ratios. Separate analyses were conducted to account for the variables days since last sleepover and location of sleepover as they did not apply to all three time-points within the study. In our first regression analysis, sex and LOS were removed. Weight, age, and previous sleepovers remained in the equation and accounted for 9.7% of the variability in cortisol values: *F* (3,124) = 5.52, *p* = .001 (Table 3).

**Table 3:** Resulting coefficients, standard errors, *t*-test and *p* values of multiple linear regression analyses with backward elimination predicting cortisol values from sex, age, weight, and LOS at each shelter.

	*Variable*	*B*	*SE B*	*t*-test	*p* value
BFAS	*Weight*	−.34	.12	−2.92	.035
	*Age*	.03	.02	2.13	.004
	*Previous Sleepovers*	−.07	.03	−2.16	.033
AHS	*Weight*	−1.60	.16	−10.11	<.001
	*Age*	.09	.03	3.17	.002
HSWM	*Weight*	−.28	.07	−4.38	<.001
DCAS	*Sex*	−3.48	1.47	−2.37	.019
	*Weight*	−.58	.12	−4.87	<.001
	*Age*	.19	.03	6.95	<.001
	*LOS*	−.05	.01	−4.68	<.001
SPCATX	*Weight*	−.38	.07	−5.62	<.001
	*Age*	.08	.02	3.71	<.001

**Notes.**

The variable “previous sleepovers” was tested as a predictor only at BFAS. Age was scaled in months.

We found that weight significantly predicted cortisol (*p* = .004), such that for each kilogram increase in weight, C/C decreased by 0.34 × 10^−6^. Age showed a contrasting effect: each year of age added 0.41 × 10^−6^ to cortisol values (*p* = .035). Additionally, the number of sleepovers significantly predicted pre-sleepover cortisol values (*p* = .033), with each sleepover reducing ratios by 0.07 × 10^−6^. Further analyses using the demographic variables of sex, age, weight, LOS, and number of sleepovers in addition to days since last sleepover (Analysis 2) and sleep location (Analysis 3) did not find any additional relationships when added to the models. These analyses are included in Table 3.

We subsequently analyzed C/C ratios from before, during, and after sleepovers using a repeated linear mixed model to detect an effect of the repeated time measure with weight, age, and number of sleepovers entered as covariates in the model. Significant differences were found among time-point means, *F* (2,50.97) = 7.34, *p* = .002. Pairwise post-hoc comparisons indicated that dogs during their sleepovers had significantly lower cortisol when compared to measurements at the shelter before (*p* = .016) and after (*p* = .015) their sleepovers. No differences were found between shelter time-points (*p* = .917). Table 4 presents BFAS C/C means and standard deviations for each morning before, during, and after sleepover. Those values are listed under Day 2, Day 3, and Day 5 to correspond to the subsequent shelter sites’ collection time-points.

**Table 4:** Mean cortisol: creatinine ratio value, standard error, *F* test statistic, and *p* value for time-points before, during, and after temporary fostering at 5 US shelters.

	Before Sleepover (Shelter)				During Sleepover (Foster)				After Sleepover (Shelter)				Test Statistics	
	1		2		3		4		5		6			
Shelter	M	SE	M	SE	M	SE	M	SE	M	SE	M	SE	*F*	*p*
BFAS			20.66^a^	0.98	18.15^a,b^	0.89			21.38^b^	1.25			7.34	.002
AHS	43.53^a^	2.92	44.51^b,c^	2.34	37.23^b^	2.03	32.80^a,c,d^	2.36	40.19^d^	2.30	38.96	1.93	5.79	<.001
HSWM	29.84	1.56	31.50^a^	1.78	26.42^b^	1.74	25.89^a,c,d^	1.69	30.85^c^	1.52	32.59^b,d^	1.70	4.81	.001
DCAS	29.06^a,b^	1.62	28.48^c,d^	1.41	24.54^a,c^	1.48	23.592^b,d^	1.72	28.06	1.49	27.60	1.55	4.13	.003
SPCATX	24.34^a^	1.52	24.67^b^	1.55	22.19	1.49	20.64^a,b,c,d^	1.49	24.49^c^	1.55	24.16^d^	1.40	3.37	.011
Overall			29.73^a^	0.79			24.92^a,b^	0.77			29.19^b^	0.75	31.71	<.001

**Notes.**

All shared lettered comparisons are significant at *p* = .05 or less except for DCAS comparison *a* (*p* = .063) and SPCATX comparisons *a* (*p* = .072) and *c* (*p* = .067).

Overall means and standard errors before, during, and after sleepover were estimated using 1-day cortisol values at BFAS and 2-day phase values at all other shelters.

### Arizona Humane Society, Humane Society of Western Montana, DeKalb County Animal Services, and SPCA of Texas

Descriptive statistics about these shelters and their dogs are included in Tables 1 and 2. Multiple linear regression analyses with backward elimination were used to identify whether sex, age, weight, or length of stay (LOS) of dogs at these each of these shelters significantly predicted their C/C ratios.

As with BFAS, the variables of weight and age remained in the equation at AHS, accounting for 33.5% of the variability in cortisol: *F* (2,231) = 59.58, *p* < .001. Weight significantly predicted cortisol (*p* < .001) such that as weight increased by one kilogram, C/C ratios decreased by 1.60 × 10^−6^. Conversely, age showed an opposite influence, such that with an increase of one year, cortisol was predicted to increase by 1.09 × 10^−6^ (*p* = .002). Table 3 includes the results of this analysis and those conducted with data from HSWM, DCAS, and SPCATX.

Cortisol values from AHS, HSWM, DCAS and SPCATX were analyzed across the six collection time-points using repeated linear mixed models to detect an effect of the repeated time measure with weight and age added into the model as covariates (except in the analysis of HSWM data where age was not a significant predictor of cortisol). Sex was added as a covariate in the HSWM analysis at LOS and DCAS.

Significant differences in cortisol values were detected at AHS, *F* (5,53.03) = 5.79, *p* < .001. Pairwise post-hoc comparisons revealed that dogs at AHS had significantly lower cortisol on their first sleepover morning compared to the morning in the shelter before their sleepovers (*p* = .016), and the second sleepover morning as compared to both mornings in the shelter before the sleepover (*p* = .011) and (*p* < .001), respectively. Additionally, in the shelter the morning after the sleepover, dogs’ cortisol values were significantly higher than in the last (or second) morning of their sleepover (*p* = .048). Table 4 includes the estimated marginal means of C/C ratios and standard errors at each collection time-point at AHS as well as at HSWM, DCAS, and SPCATX.

### Multi-shelter analyses

To uncover whether mean resting pulse rate, resting activity, longest bout of uninterrupted rest, sex, age, weight, or mean LOS significantly predicted mean C/C ratios of dogs at AHS, HSWM, DCAS, and SPCATX, a multiple linear regression analysis with backward elimination was employed. The variables of mean resting pulse, resting activity, longest bout of uninterrupted rest, mean LOS, weight, and age remained in the equation, accounting for 30.3% of the variability in these C/C ratios: *F* (6,400) = 30.39, *p* < .001.

For every one-unit increase in dogs’ mean resting pulse rate, a 0.264 × 10^−6^ increase in mean C/C was expected (*p* = .001). The proportion of time dogs spent resting predicted mean cortisol values (*p* = .01), such that with each percentage increase in rest, these cortisol: creatinine ratios increased by 0.12 × 10^−6^. However dogs’ longest bout of uninterrupted rest showed an opposite effect such that as the resting duration increased by one minute, mean C/C was predicted to decrease by 0.01 × 10^−6^ (*p* = .043). As previously shown at DCAS (Table 3), each day increase in dogs’ mean LOS, mean cortisol: creatinine ratios were predicted to decrease by 0.06 (*p* < .001). Evidenced both in the prior analyses of AHS and SPCATX data, dogs’ weight and age significantly predicted mean cortisol values (*p* < .001), such that with each one-kilogram increase in weight, mean C/C ratios increased by 0.55 × 10^−6^; and with each year increase in age, a 1.01 × 10^−6^ increase in mean C/C was expected (*p* < .001).

With all participating shelters, mean cortisol values from before, during, and after sleepovers were calculated and analyzed using a linear mixed model to detect an effect of the repeated time measure. The covariates of weight, age, and mean LOS were added into the model based on the previous individual analyses of the shelters in which these variables were found to be of statistical significance. Significant differences were found among these phases, *F* (2, 209.04) = 31.71, *p* < .001. Dogs had significantly lowered mean cortisol values during the sleepover as compared to before the sleepover in the shelter (*p* < .001) and after (*p* < .001), with no difference in the before and after comparison (*p* = .799). Table 4 presents the estimated marginal means and standard deviations for before, during, and after sleepover. Those values are listed under Day 2, Day 4, and Day 6.

**Figure 1:**
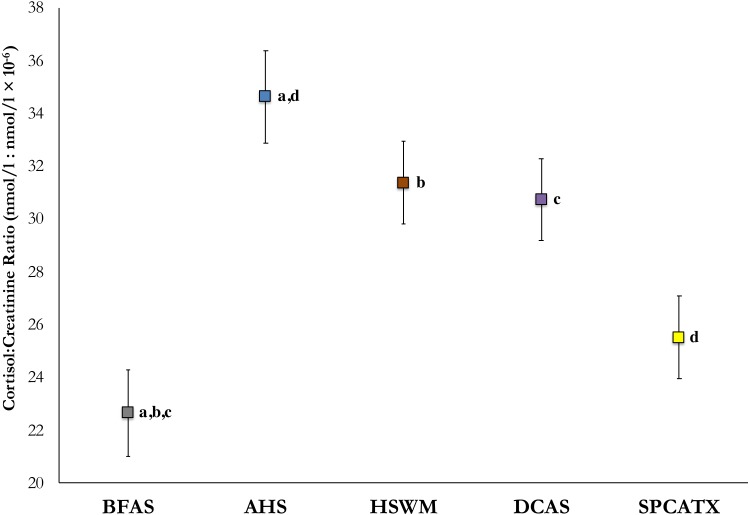
Estimated marginal means of average in-shelter cortisol: creatinine ratio values and standard errors at five US shelters. All shared lettered comparisons are significant at *p* < .05.

To detect an effect of shelter, mean cortisol values from in-shelter collections (before and after sleepover) at all five participating sites were analyzed using a linear mixed model, with weight and age added into the model as covariates. With these values, significant differences were observed, *F* (4, 196.41) = 7.14, *p* < .001. In post-hoc comparisons, dogs at BFAS were found to have significantly lower mean cortisol values than dogs at AHS (*p* < .001), HSWM (*p* = .003), and DeKalb (*p* = .004) with dogs at SPCATX having lower mean cortisol than dogs at AHS (*p* = .002). Figure 1 includes the estimated marginal means of C/C ratios and standard errors for each shelter.

Dogs’ average resting pulse and longest bouts of rest before, during, and after their sleepovers were analyzed using linear mixed models to detect any effect of phase, shelter, or a shelter-by-phase interaction, with the covariates of weight, age, and mean LOS added into the model. No main effects of phase, shelter, or shelter-by-phase interaction were found with the dogs’ pulse rates at rest.

**Figure 2:**
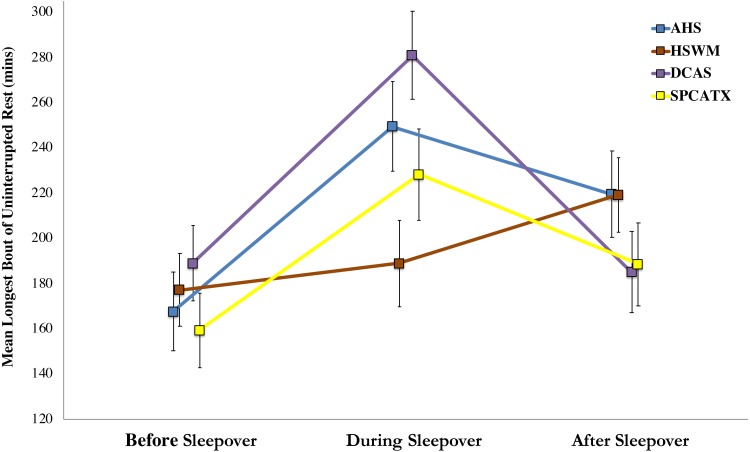
Estimated marginal means of dogs’ longest bouts of uninterrupted rest and standard errors for four US shelters before, during, and after dogs’ sleepovers. Shared letter comparison is significant at *p* < .05.

For the dogs’ longest bouts of rest, the shelter-by-phase interaction was significant, *F* (6, 183.04) = 3.13, *p* = .006, indicating that dogs at the five shelters differed in their resting bouts before, during, or after their sleepovers. Upon further inspection, only a single, significant post-hoc comparison was found: dogs at DCAS had longer bouts of rest while away from the shelter than dogs at HSWM (*p* = .007). A more robust main effect of phase was found, *F* (2, 193.51) = 16.99, *p* < .001, demonstrating dogs’ resting bouts differed during the study. Post-hoc comparisons revealed that dogs had their longest bouts of uninterrupted rest during sleepovers (*p* < .001). Resting bouts in the shelter after sleepovers were longer than before they left (*p* = .012) but shorter than during sleepovers (*p* = .010). No main effect of shelter was found, indicating that dogs’ longest bouts of rest did not differ by study site. Figure 2 includes the estimated marginal means of dogs’ longest bouts of uninterrupted rest and standard errors for each shelter before, during, and after dogs’ sleepovers.

## Discussion

Our hypothesis, that temporary fostering of shelter dogs for one and two nights in volunteers’ homes would reduce stress was supported: dogs’ cortisol: creatinine ratios were significantly decreased during their sleepovers. However, C/C ratios did rebound to pre-sleepover values upon return to the shelter. We found at each shelter that dogs of greater weight had lower C/C ratios, supporting previous findings by Zeugswetter et al. (2010). With the exception of one shelter (HSWM), dogs’ cortisol values increased with age in our study, which has previously been shown in Rothuizen et al. (1993). In the four shelters in this study where health-monitoring collars were used, higher average resting pulse rates and more time spent resting were associated with higher cortisol, whereas the longest bout of uninterrupted rest was related to lower C/C values. Dogs had their longest bouts of rest during their sleepovers, followed by in the shelter after their sleepovers. At BFAS, where a sleepover program was in place prior to our study, the number of sleepovers a dog had previously experienced was correlated with lower cortisol. Additionally, we were able to detect differences between shelters when comparing their average in-shelter cortisol values. These between-shelter differences were, in some cases, larger than the reduction provided by temporary fostering.

C/C ratios have been utilized in previous studies to assess the stress of kenneled and shelter dogs (Beerda et al., 1999a; Beerda et al., 1999b; Hiby, Rooney & Bradshaw, 2006; Stephen & Ledger, 2006; Titulaer et al., 2013). As a measurement of welfare, urinary cortisol provides a particularly useful reflection period of approximately two-to-four hours (Schatz & Palme, 2001) and allows for a non-invasive approach in assessing the physiological stress response of dogs. While previous studies have found elevated cortisol levels in shelter dogs compared to groups of dogs in homes (Sandri et al., 2015; Dudley, Schiml & Hennessy, 2015), little is known about the effect of living condition on C/C ratios in the same dogs. Rooney, Gaines & Bradshaw (2007) found that urinary cortisol values of dogs entering military training kennels were not only higher than the same dogs’ home values; but dogs that had previously spent time in the kennel environment during a habituation program had significantly lower cortisol than those without such experience, and this difference persisted across the initial ten days in the kennels. Accounting for differences in cortisol responding between dogs, Rooney, Gaines & Bradshaw (2007) provided compelling evidence of the stress undergone by dogs when transitioning from a home to a kenneling environment and how prior experience may impact stress responding.

Only one prior study (Fehringer, 2014) has examined the effect of changes in living conditions in the opposite direction from Rooney, Gaines & Bradshaw (2007); that is to say, measuring the physiological and behavioral effects of placing dogs from animal shelters into homes. Fehringer (2014) found that placement in homes lowered dogs’ salivary cortisol values compared to in-shelter values and that the dogs’ cortisol concentrations steadily declined over the first three days of foster care. Although saliva samples used in cortisol analyses are more sensitive to immediate stressors the dogs have experienced (Beerda et al., 1996) than the urine collection method we utilized, nonetheless these results parallel our findings in suggesting that the transition and placement in a home environment is likely less stressful than shelter living.

To our knowledge, ours is the first study to examine the impact of temporary fostering on the cortisol response of dogs awaiting new homes. While adoption programs, such as those described in Fehringer (2014) and Mohan-Gibbons et al. (2014), do involve the fostering of dogs living in shelters, these programs housed dogs with volunteers until placement or study completion. Conversely, temporary fostering, or sleepovers, in this study are departures of one-to-two days with intended return to the shelter. Thus, sleepovers should be viewed primarily as a form of enrichment for shelter-living dogs.

In our analysis of dogs’ heart rate and resting data, we found a positive correlation between resting heart rate and cortisol, which had previously been observed in Beerda et al. (1997), affirming this relationship and demonstrating the ability of commercially available health-monitoring collars to capture this measure. More difficult to understand is the positive relationship between proportion of time spent resting and C/C ratio values. One explanation may be found by considering the behaviors that were likely included in this measure of rest. Activity was collected by 3-D accelerometers that are sensitive to movement. With this particular monitoring device, a dog could be lying down, sitting, standing or even slightly moving and still be considered resting (Asaf Dagan, personal communication, March 19, 2018). In a contemporaneous study (Gunter, 2018) certain postures, such as a dog lying down with its head down, were found to be associated with lower cortisol and metanephrine values, while standing was associated with higher levels. Previously, Hekman, Karas & Dreschel (2012) also found that a dog resting with its head down was correlated with lower salivary cortisol levels. It is possible therefore that, while these collars are detecting a lack of movement by the dogs, the behaviors involved in the “at rest” measure may correspond to differing physiological states.

Conversely, dogs’ longest period of uninterrupted rest as measured by the health-monitoring collars was not only negatively associated with their cortisol response, but the longest periods of rest were observed during the sleepovers, and rest bouts upon return to the shelter were longer than those before the sleepover. Considering that resting bouts were nearly six hours in length, on average, in the foster home, it is most likely these dogs were in sleep or near sleep states; and this measure, unlike our proportion of rest activity, was a better measure of rest because it is unlikely that dogs would remain sitting or standing for such a long period of time. Taken together with previous findings regarding the resting behavior of shelter and owned dogs (Owczarczak-Garstecka & Burman, 2016; Morrison et al., 2013), our results suggest that dogs may be experiencing a sleep deficit in shelters, which may explain the longer bouts of rest recorded while in the foster home. Differences between shelters in these resting bouts may provide insight into the level of stress dogs are experiencing while in the shelter. Post-sleepover, the longer bouts of uninterrupted rest that we measured are consistent with anecdotal shelter staff reports of more restful behavior displayed in-kennel by dogs after their sleepovers.

We also noted significant differences between shelters in dogs’ in-shelter cortisol values (averaged from collections taken before and after dogs’ sleepovers) at our five participating sites. It has previously proven difficult to compare cortisol values across shelters, because data from different shelters are presented in disparate studies, with differing methods of collection and study participants varying in important parameters (e.g., age and weight). In the present study, researchers and methods were consistent across shelters. Furthermore, variables known to affect cortisol values were included in our analysis. Our findings correspond with those of McCobb et al. (2005) with cats. McCobb et al. found that feline C/C ratios differed significantly between shelters, such that those that provided greater environmental enrichment had cats with lower C/C ratio values than cats in more traditional shelters. Considering the diversity of open- and limited-admission shelters in our study where annual intake ranged from under 1,000 to over 6,000 dogs, it is certainly possible that as yet unstudied factors, such as dog density, kennel conditions, and/or enrichment programs could contribute to the overall welfare of shelter dogs.

While significant physiological benefits were observed for the dogs at each shelter, the shelter with the highest in-shelter cortisol values (AHS) benefited the greatest from the intervention, with nearly a one-quarter reduction in C/C ratios. Conversely, cortisol responding of dogs at the shelter with lowest baseline cortisol values (BFAS) decreased by only 12% when staying overnight in a fosterer’s home. While this difference in benefit from the sleepover could be attributed to differing levels of stressors at these shelters, other variables may play a role. It is possible that had the sleepover continued for a second night, dogs from BFAS may have experienced as great a reduction as was observed at all other shelters in the study where two-night sleepovers were tested. However, the results from SPCATX, where the dogs also had lower initial in-shelter C/C ratios and experienced two-day sleepovers, showed a lessened effect of the sleepover, contradicting this hypothesis.

As demonstrated by the shelters within our study, C/C values at the end of study enrollment were not significantly lower (or higher) than initial in-shelter values, suggesting that the benefits of the sleepover were short-lived. This result is consistent with shorter (15 and 30 min) human interaction interventions that have been investigated within animal shelters (Willen et al., 2017). Nonetheless, evidence from BFAS indicates that dogs that left the shelter on more sleepovers had lower cortisol levels. Although it should be acknowledged that this effect on cortisol was small and other characteristics of the dog likely play a role, it is a finding that mirrors the habituation effect observed by Rooney, Gaines & Bradshaw (2007). Thus, we recommend future studies should investigate the potential benefits of repeated sleepovers for longer stay shelter dogs.

While interventions such as the one described here, or out-of-kennel interactions of varying length and activities (Coppola, Enns & Grandin, 2006; Menor-Campos, Molleda-Carbonell & López-Rodríguez, 2011; Shiverdecker, Schiml & Hennessy, 2013) may provide relief for dogs in shelters, it is possible that shelter design or daily procedures may afford more sizeable reductions in stress levels. With the exception of the significant cortisol decrease observed at AHS during sleepovers, differences between shelters in their in-shelter C/C values, such as with BFAS or SPCATX and any of the other shelters, were greater in magnitude than the reductions obtained during the sleepover intervention itself. Further studies in which environmental factors are manipulated between and within dogs at the same facility may elucidate how routine husbandry, care, or other management procedures may have a greater impact on shelter dog welfare than more temporary enrichment interventions.

One limitation in our study is that not all dogs were eligible for participation, particularly fearful dogs that were not able to walk on leash for urine collection or dogs deemed unsafe by shelter staff. Previous findings from Hiby, Rooney & Bradshaw (2006) suggest that dogs displaying more startle and ambulatory behaviors initially in the shelter have higher cortisol levels; therefore, it is possible that transitioning to a new foster environment for these types of dogs may not provide the reduction in cortisol or increases in resting bouts that we observed. Additionally, urine sampling was conducted in the morning, not long after waking; and as such, C/C levels may not reflect a dog’s 24-hour experience in the shelter. Research by Beerda et al. (1996) has suggested morning sampling may yield higher C/C ratios compared to the afternoon, although more recent work with a larger sampling of owned dogs has failed to replicate this effect (Zeugswetter et al., 2010). Thus, it is possible a later day urine collection during our study may have found a less robust effect of living condition, particularly if shelter environment was perceptively less stressful for dogs in the evening hours.

## Conclusions

This study demonstrates that shelter dogs’ urinary cortisol concentrations systematically decrease and bouts of uninterrupted rest increase when the dogs are placed into temporary foster homes, as compared to in-shelter values obtained prior to and after the sleepover. This reduction in stress, observed at all five participating shelters, varied in its magnitude and was lost once dogs returned to the shelter, although bouts of restful behavior post-sleepover were longer than pre-sleepover durations. Dogs’ cortisol values measured in-shelter differed between shelters; and in some cases, these differences were larger than the reduction provided by the temporary fostering intervention. In total, these findings suggest that while a reprieve from the shelter is positively impactful for the welfare of companion dogs, mitigating the stressors present in kenneling conditions that are likely contributing to higher stress responding should also be addressed to improve the overall welfare of shelter-living dogs.

## Additional Information and Declarations

### Data Availability

The following information was supplied regarding data availability: (2018). Evaluating the Effects of a Temporary Fostering Program on Shelter Dog Welfare [Data set]. University Libraries, Virginia Tech https://doi.org/10.7294/w776-cc07.
